# Effect of corn supplementation on purine derivatives and rumen fermentation in sheep fed PKC and urea-treated rice straw

**DOI:** 10.1007/s11250-018-1636-1

**Published:** 2018-06-14

**Authors:** Osama Anwer Saeed, Awis Qurni Sazili, Henny Akit, Abdul Razak Alimon, Anjas Asmara B. Samsudin

**Affiliations:** 1 Department of Animal Science, Faculty of Agriculture, Universiti Putra Malaysia, 43400 Serdang, Selangor Malaysia; 2 Department of Animal Science, Agriculture Faculty, University of Anbar, Ramadi, Iraq

**Keywords:** Corn, Palm kernel cake, Purine derivatives, Nitrogen retention, Microbial population, Rumen fermentation

## Abstract

This study investigated the effect of different levels of corn supplementation as energy source into palm kernel cake–urea-treated rice straw basal diet on urinary excretion of purine derivatives, nitrogen utilization, rumen fermentation, and rumen microorganism populations. Twenty-seven Dorper lambs were randomly assigned to three treatment groups and kept in individual pens for a 120-day period. The animals were subjected to the dietary treatments as follows: T1: 75.3% PKC + 0% corn, T2: 70.3% PKC + 5% corn, and T3: 65.3% PKC + 10% corn. Hypoxanthine and uric acid excretion level were recorded similarly in lambs supplemented with corn. The microbial N yield and butyrate level was higher in corn-supplemented group, but fecal N excretion, T3 has the lowest level than other groups. Lambs fed T3 had a greater rumen protozoa population while the number of *R. flavefaciens* was recorded highest in T2. No significant differences were observed for total bacteria, *F. succinogenes*, *R. albus*, and methanogen population among all treatment. Based on these results, T3 could be fed to lambs without deleterious effect on the VFA and N balance.

## Introduction

The cost of conventional feed ingredients for livestock, coupled with global climate change, has made farmers in the tropical countries to go for low-cost feed ingredients such as roughages. In some tropical countries, ruminants are fed on agricultural by-products like palm kernel cake (PKC). This ingredient has moderate crude protein (CP) content, and utilization of PKC in sheep is highly dependent on the rumen microbial activity to produce fatty acids and microbial protein. Like other ruminants, sheep are able to utilize lignocellulosic materials and convert them to animal products of high nutritional value, such as meat, milk, wool/fur, hide, and manure. This is due to the presence of a dense and diverse rumen microbial population belonging to a different group of flora and fauna (Agrawal et al. 2014) as well as rumen microbial protein which is important for amino acid absorption. Urinary purine derivatives (PD) excretion is used to assume the rumen microbial protein synthesis. The principle is that duodenal purine bases, as a microbial indicator, are efficiently absorbed, and their derivatives excreted mainly via kidney. The ratio of PD in urine can be used to predict microbial N flow (Belenguer et al. 2002).

The literature indicates an increase in energy intake and essential nutrients such as amino acids could have a positive effect on ruminal digestion that would fasten the emptying process of the ruminal contents and create a space for more feed consumption. Therefore, the present study was undertaken to determine the utilization of nitrogen (N) and to validate the potential value of urinary purine derivatives, rumen fermentation, and microorganism populations in Dorper sheep fed with corn supplementation, in addition to PKC as basal diet.

## Materials and methods

### Experimental site

Animal experimentation was carried out at Animal Farm of Universiti Putra Malaysia. The farm is located in Serdang, Selangor, Malaysia (3° 2′ 0″ North, 101° 43′ 0″ East). The animal experiment was conducted in accordance with the procedure of Institutional Committee on Animal Use Ethics (Approval No. R064/2016).

### Animal, housing, and diet

Twenty-seven Dorper lambs (body weight = 15 ± 0.59 kg; aged 6 months) were used. The experimental period consisted of 15 days of dietary adaptation and 120 days for sampling and data collection. Lambs were housed in individual pens and had free access to water. The animals were randomly allocated to three dietary treatments as follows: T1: 75.3% PKC + 0% corn, T2: 70.3% PKC + 5% corn, and T3: 65.3% PKC + 10% corn. All diets were approximately isonitrogenous and were formulated to meet the metabolizable energy for growing requirements of lambs weighing 15 kg (NRC 2007) (Table 1). The diets were offered two times daily at 0800 and 1600 h and provided together with the roughage (urea-treated rice straw) and concentrate (20:80).

**Table 1:** Ingredients and chemical compositions of different levels of corn into PKC–urea-treated rice straw (DM basis)

Item	Levels of corn (%)		
	T1	T2	T3
Urea-treated rice straw	20	20	20
PKC	75.3	70.3	65.3
Protected fat (Megalac)	3	3	3
Corn	0	5	10
CaCO_3_	1	1	1
NaCl	0.5	0.5	0.5
Vitamin premix	0.2	0.2	0.2
Total	100	100	100
Chemical composition			
DM	91.78	91.66	91.55
Ash	13.80	12.72	12.74
OM	86.19	87.27	87.26
CP	15.42	14.88	14.09
EE	5.3	5.1	4.33
CF	26.6	24.50	20.83
NDF	62.36	60.06	55.66
ADF	45.60	40.96	37.30
ADL	6.56	6.10	5.43
Hemicellulose	16.76	19.10	18.36
Cellulose	39.03	34.86	31.86
NFE	40.44	41.11	48.39
ME MJ/kg DM	7.36	8.23	8.92

Vitamin premix; vitamin A 10,000,000 IU; vitamin E 70,000 IU; vitamin D 1,600,000 IU. *DM* dry matter, *OM* organic matter, *CP* crude protein, *EE* ether extract, *CF* crude fiber, *NDF* neutral detergent fiber, *ADF* acid detergent fiber, *ADL* acid detergent lignin, *NFE* nitrogen-free extract, *ME* metabolizable energy

### Urine collection

Urine samples were collected on days 3 and 7 from individual animals and were filtered using four layers of cheesecloth following the method described by Balcells et al. (1992). Approximately 10% of H_2_SO_4_ was dropped (pH of the urine < 3) to avoid volatilization of ammonia, and the urine samples were stored at − 20 °C until analysis.

### Chemical analysis

Samples of feeds and fecal were analyzed according to the AOAC (1990). Nitrogen content was calculated as crude protein (CP) ÷ 6.25. Nitrogen retention was computed from dietary N intake (NI) less total nitrogen output (fecal N [FN] and urinary N [UN] losses) by Katsande et al. (2016).

$$ {\displaystyle \begin{array}{c}\mathrm{Nitrogen}\ \mathrm{retention}\ \left({\mathrm{g}}^{\mathrm{d}-1}\right)=\mathrm{NI}\hbox{--} \left(\mathrm{FN}+\mathrm{UN}\right)\\ {}\mathrm{Apparent}\ \mathrm{digestibility}\ \left(\%\right)=\mathrm{NI}\hbox{--} \left(\mathrm{FN}+\mathrm{UN}\right)\ \mathrm{NI}\times 100\end{array}} $$

Purine derivatives, such as allantoin, uric acid, xanthine, and hypoxanthine concentrations in urine samples, were measured according to Balcells et al. (1992). The samples were examined using high-performance liquid chromatography (HPLC) (Agilent 1100 Series HPLC System Agilent Technologies, USA) by two 4.6 mm × 250 mm C-18 reverse-phase column (Spherisorb), and the effluent was monitored at 205 nm.

Determination of volatile fatty acids (VFA) in ruminal fluid was as described previously (Cottyn and Boucque 1968). The pH of the rumen fluid was assessed with the aid of a Mettler-Toledo pH meter (Mettler-Toledo, Ltd. England). Ammonia nitrogen (NH_3_-N) in ruminal fluid was determined in accordance with Parsons et al. (1984).

### Microbial population assay

Rumen fluid samples were collected from animals after slaughter. Then, the samples were squeezed through four layers of cheesecloth and kept frozen (− 20 °C) for further analysis. Total bacterial DNA was extracted using the QIAamp® DNA mini stool kit (Qiagen, Hilden, GmbH) according to the manufacturer’s protocol. DNA was quantified using a Nanodrop ND-1000 spectrophotometer (Thermo Fisher, Waltham, MA) and used as a template for setting up all polymerase chain reactions (PCRs).

Bacteria populations were quantified using the real-time PCR machine (BioRad, USA), primers were chosen from previously published sequences that demonstrated species-specific amplification. The nucleotide sequences of the primers used in this study are shown in Table 1. The different microbial groups, such as total bacteria, methanogens, *Fibrobacter succinogenes*, *Ruminococcus albus*, *Ruminococcus flavefaciens*, and protozoa were determined in the samples using quantitative real-time PCR according to Ahmed et al. (2017).

### Statistical analysis

Data were analyzed with a completely randomized design using the general linear model procedure of SAS 9.4 (SAS Inst. Inc., Cary, NC, USA). All the multiple comparisons among means were determined using Duncan’s multiple range test (*P* < 0.05).

## Results

### Purine derivatives

There were highly significant differences (*P* < 0.001) in the purine derivatives in sheep. Diet influenced (*P* < 0.05) microbial N yield, true microbial protein, digestible microbial true protein, and digestible organic matter (DOM) in rumen of the sheep fed on T2 and T3 (Table 2). However, the excretion of uric acid, hypoxanthine, and the efficiency of microbial protein supply were similar among dietary treatments.

**Table 2:** Urinary purine derivatives excretion at different levels of feed intake of corn

Parameter	T1	T2	T3	SEM	*P* value
Allantoin excreted (mmol/W^−0.75^)	4.89^b^	5.26^b^	7.37^a^	0.27	0.001
Uric acid excreted (mmol/W^−0.75^)	1.42	1.61	1.75	0.09	0.36
Hypoxanthine excreted (mmol/W^−0.75^)	1.93	1.57	1.73	0.07	0.11
Xanthine excreted (mmol/W^−0.75^)	0.38^c^	1.03^b^	1.16^a^	0.07	0.001
Purine derivative excreted (mmol/W^−0.75^)	8.5^b^	9.2^b^	11.4^a^	0.35	0.001
Microbial N yield (MNY; g day^−1^)	6.89^b^	8.83^a^	9.07^a^	0.35	0.05
Microbial true protein (MTY; g day^−1^)	34.5^b^	44.2^a^	45.4^a^	1.75	0.05
Digestible microbial true protein (g day^−1^)	29.3^b^	37.5^a^	38.6^a^	1.49	0.05
Digestible organic matter in rumen (g day^−1^)	0.23^b^	0.23^b^	0.31^a^	0.003	0.05
E_mns_ (g day^−1^ DOMR)	32.3	32.3	31.8	0.35	0.81

T1: (75.3% PKC + 0% corn), T2: (70.3% PKC + 5% corn), T3: (65.3% PKC + 10% corn). ^a,b,c^Means in the same row with different superscripts are significantly different

### Nitrogen balance

Nitrogen intake was recorded the highest for lambs fed with T3 diet (Table 3). The highest (*P* < 0.05) fecal N excretion was observed in T2 diet (10.9 g/day) showing that N that passed to the lower gut was not efficiently digested than another treatment group. Lambs fed with T2 and T3 had higher (*P* < 0.05) urine N excretion than T1. Nitrogen apparent absorption and N retention did not differ among T1 and T2, but T3 had a higher (*P* < 0.001) of the two parameters.

**Table 3:** Daily nitrogen balance of Dorper sheep fed on different levels of corn

Item	T1	T2	T3	SEM	*P* value
Intake (g/day)	22.9^b^	22.2^b^	28.8^a^	0.30	0.001
Excretion (g/day)					
Fecal	10.67^a^	10.94^a^	8.70^b^	0.26	0.05
Urinary	0.04^b^	0.07^ab^	0.08^a^	0.006	0.05
Total	8.21	7.84	6.71	0.52	0.49
Apparent absorption					
g/day	12.9^b^	11.2^c^	20.1^a^	0.32	0.001
Of intake %	52.7^b^	48.2^c^	67.9^a^	0.628	0.001
Retention					
g/day	14.7^b^	14.3^b^	22.0^a^	0.31	0.001
Of intake %	61.9^b^	62.9^b^	75.2^a^	0.485	0.001

T1: (75.3% PKC + 0% corn), T2: (70.3% PKC + 5% corn), T3: (65.3% PKC + 10% corn). ^a,b,c^Means in the same row with different superscripts are significantly different

### Rumen fermentation and microbial population

The supplementation of corn in diets did not affect the ruminal fluid concentrations of NH3-N and pH. There were significant differences of VFA for the lambs in this study (Table 4). The isopropionate and isobutyrate levels were similar across treatments. Acetate was higher (*P* < 0.05) while propionate was lower (*P* < 0.05) for the lambs fed with T2 diet. Butyrate was higher (*P* < 0.05) in lambs fed with corn-supplemented diet. The acetate:propionate ratio of the lambs in this study differed among dietary treatments with T2 and T3 being higher (*P* < 0.05) compared to T1. No significant differences were observed for total bacteria, *F. succinogenes*, *R. albus* and methanogen population among all treatment (Table 5). T3 had a greater protozoa population (*P* < 0.05) while the number of *R. flavefaciens* was recorded highest in T2.

**Table 4:** Means of NH_3_-N, pH, and production of VFA (mmol/ml) in vivo fermentation of different corn supplementation

Parameters	T1	T2	T3	SEM	*P* value
NH_3_-N (mg/100 ml)	6.59	6.24	7.00	0.43	0.79
pH	6.90	6.96	6.80	0.04	0.36
Total VFA (mmol/ml)	18.1^b^	14.1^b^	30.8^a^	2.47	0.01
Acetate	48.8^b^	52.3^a^	48.5^b^	0.73	0.05
Propionate	32.8^a^	20.7^b^	24.1^b^	1.72	0.01
Isopropionate	3.25	3.23	3.44	0.16	0.85
Butyrate	12.9^b^	16.7^a^	17.2^a^	0.76	0.05
Isobutyrate	7.75	6.98	6.85	0.25	0.38
C2:C3	1.53^b^	2.56^a^	2.16^ab^	0.15	0.05

T1: (75.3% PKC + 0% corn), T2: (70.3% PKC + 5% corn), T3: (65.3% PKC + 10% corn). ^a,b^Means in the same row with different superscripts are significantly different

**Table 5:** Effect of dietary treatments on microbial population (copies/ml) in the rumen of sheep

Item	Diets				
Species	T1	T2	T3	SEM	*P* value
Total bacteria (× 10^10^)	10.1	10.1	10.0	0.08	0.88
*F. succinogenes* (× 10^9^)	5.47	5.11	5.34	0.09	0.39
*R. albus* (× 10^6^*)*	6.86	6.74	7.13	0.11	0.35
*R. flavefaciens* (× 10^7^)	4.75^b^	5.27^a^	4.99^ab^	0.17	0.05
Methanogenic archea (× 10^9^)	5.05	4.93	4.55	0.10	0.54
Total protozoa (× 10^5^)	3.33^b^	3.37^b^	4.24^a^	0.18	0.05

T1: (75.3% PKC + 0% corn), T2: (70.3% PKC + 5% corn), T3: (65.3% PKC + 10% corn). ^a,b^Means in the same row with different superscripts are significantly different

## Discussion

### Purine derivatives

In ruminants, most of the PD excreted in urine comes from the partial metabolism of microbial nucleic acid absorbed into the duodenum. However, there was a significant difference of the urinary PD among treatments where the PD concentration increased as the level of corn supplemented in the diet increased. In this regard, hypoxanthine and uric acid excretions were not affected by the corn diets. The microbial N yield, true microbial protein, digestible microbial protein, and DOM in rumen were higher in sheep T2. These results are consistent with the findings of Chanjula and Pongprayoon (2012) who had worked on in vivo degradability of feeding rubber seed kernel and PKC in goats. Dietary treatment in the present study did not influence the efficiency of microbial N synthesis with the values ranging from 31.8 to 32.3 g day^−1^DOMR. The lack of changes in Emns might be due to various factors such as the concentration and sources of nitrogen and carbohydrates. Furthermore, Singh et al. (2007) reported that the excretion of urinary PD was positively correlated with the level of feed intake. A higher excretion of allantoin and xanthine clearly indicates an enhanced microbial protein synthesis in T2 and T3 due to the significant correlation between allantoin and xanthine excretions and the level of nucleic acid infused into the abomasum (Chen et al. 1990). Variation in the efficiency of microbial N synthesis may relate to the level of feeding on the rumen fermentation where a higher feed intake could manipulate the pH level in the rumen (Ribeiro et al. 2017).

The energy and protein contents in the diet seem to be sufficient for ideal microbial growth, and the protein resistance to microbial degradation may constrain the synthesis of microbial protein. The urine samples confirmed the high ability of cattle to oxidize absorbed purine bases to non-reutilizable PD (Chen et al. 1990).

Since microbial protein synthesis is dependent on fermentable OM and NH3-N supply, it is anticipated that the efficiency of synthesis will decrease with the reduction of feed intake.

### Nitrogen balance

Corn supplementation has increased N intake in T3. Tedeschi et al. (2003) explained that the increase in N retention is due to improved N digestibility. Lambs fed on T3 reduced N excretion in fecal and urine in the present experiment. This finding conflicted with Wanapat et al. (2000) who observed a higher NDF and ADF digestibility with the increase in N levels in the ration. The N absorption was not similar between treatments which tended to increase in T3 compared to the rest. Utilization of N is always referred to N excretion and N retention which reflect the variances in N metabolism, which was the most significant index of the protein nutrition condition of ruminants (Firkins et al. 2007). In this study, a lower N retention was due to the excretion of excess N in the urine and fecal matter. The high amount of protein consumed resulted in high N retention in the body which could be utilized by the animals. Sarwar et al. (2003) proved that the ability to retain N is dependent on N intake and the quantum of fermentable carbohydrate of the diet.

### Rumen fermentation

The concentration of NH3-N is in line with Ludden et al. (2002). The level of ruminal NH3-N in the present study was sufficient to maintain fermentation process as shown by the consistencies in VFA levels and fiber digestibility. Both were within the optimal range of 2.0 to 5.0 mg/dl that would maintain microbial to growth. The ruminal pH value has an important function on the concentration of dry feed matter digestibility (DMD) and on the protozoa (ciliate) survival and development (Voia et al. 2014). In this study, ruminal pH did not change and remained within normal range among treatments. This could be the result of the presence of protozoa in the rumen which aids the stability of the rumen pH in animal fed diets that are rich in starch and reduced the redox potential of rumen digesta (Nagaraja 2016).

The reduction in the molar proportion of acetate to propionate ratio was consistent with the supplementation of corn in T2 and T3 diets. In this study, the concentration of acetate was higher in T2 and T3 and may even reach 75% of the total VFA when the diet is composed of forage (Li et al. 2014), which is responsible for higher energy production. The highest production of butyrate was obtained in T2. Butyrate has been correlated with protozoa abundance, and this result is in line with Shen et al. (2017) who explained the reduction of butyrate reflected a decline in the number of protozoa as a butyrate producer.

### Rumen microbiology

The concentration of *R. flavefaciens* was highest in T2. *R. flavefaciens* plays a role in the digestion of CF, and the corn supplementation encourages the breakdown of fibrolytic bacteria in the rumen. Furthermore, it was observed that the total number of protozoa increased as supplementation of corn increased (T3), and this is similar to what was observed by Valente et al. (2016). The increase in *R*. *flavefaciens* population in the rumen probably is not related to the reduction of protozoa but is associated with the quantity of fiber content in the diet. Fibrolytic species, such as *R. albus*, *R. flavefaciens*, and *F. succinogenes* could digest fiber faster and to a greater extent. In fact, these species even digest crystalline cellulose more actively than ruminococcal species as reported by Koike and Kobayashi (2001). On the other hand, it is known that the physical disruption of forage material stimulates microbial access, colonization, and fermentation in the rumen (Pan et al. 2003).

A higher number of protozoa were beneficial for lambs as it could metabolize significant amounts of lactate to propionate thus restricted the risk of acidosis (Kongmuna et al. 2009). Therefore, the T2 and T3 may have higher in DNA copy number of ciliate protozoa population, but the protozoa does not have an effect on reducing *F. succinogenes*, *R. albus*, and *R. flavefaciens* or other bacteria species.

## Conclusion

The results of this study indicate that the supplementation of corn into PKC–urea-treated rice straw had no effects on uric acid, hypoxanthine, total N excretion, NH_3_-N, pH, and total bacteria. Taken together, the study concludes that an optimal level of 5% corn and PKC levels of 70.3% in the diet can be fed to lambs without compromising rumen metabolism.
